# Enhancing emergency care for older persons: the role and impact of the electronic Frailty Index

**DOI:** 10.1007/s11357-024-01472-x

**Published:** 2025-01-13

**Authors:** Dana Shiffer, Antonio Desai, Matteo Cesari, Elena Generali, Massimiliano Greco, Gabriele Savioli, Mauro Giordano, Antonio Voza

**Affiliations:** 1 Department of Emergency Medicine, IRCCS Humanitas Research Hospital, Rozzano, Milan Italy; 2 Department of Biomedical Sciences, Humanitas University, Pieve Emanuele, Milan, Italy; 3 Department of Clinical Sciences and Community Health, University of Milan, Milan, Italy; 4 Department of Anesthesiology and Intensive Care, IRCCS Humanitas Research Hospital, Rozzano, Milan Italy; 5 Emergency Department, IRCCS Policlinico San Matteo, Pavia, Italy; 6 Department of Advanced Medical and Surgical Sciences, University of Campania “L. Vanvitelli”, Naples, Italy

**Keywords:** Frailty, Geriatric, Electronic Frailty Index, Risk stratification, Frailty score

## Abstract

As the elderly population expands, enhancing emergency department (ED) care by assessing frailty becomes increasingly vital. To address this, we developed a novel electronic Frailty Index (eFI) from ED health records, specifically designed to assess frailty and predict hospitalization, in-hospital mortality, ICU admissions, and 30-day ED readmissions. This retrospective, single-center study included patients 65 years old or older who presented to the ED of IRCCS Humanitas Research Hospital in Milan, Italy, between January 2015 and December 2019. Frailty was assessed using a novel electronic Frailty Index (eFI), based on the cumulative deficit model, incorporating 45 health deficits to quantify frailty. Patients were divided into four quartiles based on eFI scores to explore the association between frailty levels and adverse outcomes, including hospitalization, in-hospital mortality, ICU admission, and 30-day ED readmission. The study included 21,537 patients (mean age 77.4, 50.7% males). The median eFI score was 0.16. Hospitalization rates rose significantly with frailty, from 20% in the least frail quartile to 43% in the most frail. Similarly, in-hospital mortality and ICU admissions increased markedly with higher eFI scores, with mortality rates climbing from 0.44 to 5.0% across quartiles. The 30-day ED readmission rates significantly rose from 9.9 to 19.8%. For every 0.01 increase in eFI score, the odds of hospitalization, in-hospital mortality, ICU admission, and 30-day ED readmission significantly increased (*P* < 0.0001). Specifically, the adjusted odds ratios (OR) for hospitalization, in-hospital mortality, ICU admission, and ED readmission rose to 3.55, 14.15, 4.70, and 2.22, respectively (*P* < 0.0001), in the most frail compared to the least frail quartile. The integration of the eFI into ED settings can enable more precise risk stratification and resource allocation, significantly improving patient management and healthcare delivery for older persons in these urgent care contexts.

**Supplementary Information:**

The online version contains supplementary material available at 10.1007/s11357-024-01472-x.

## Introduction

The global population of individuals aged 65 and above is expected to reach 20% by 2050 [1]. With aging, the prevalence of multi-dimensional health challenges such as chronic conditions and cognitive impairments increases. Among these, frailty, a geriatric syndrome characterized by a decrease in physiological resilience, increases the risk of severe outcomes such as disability, hospitalization, and mortality [2, 3], affecting an estimated 10% of the geriatric population [4].

While the clinical frailty scale (CSF) has been a valuable routine tool in routine assessments across several countries, its broad categorization and reliance on subjective evaluations can limit its precision and adaptability in emergency settings [5, 6]. Unlike the CFS, the Frailty Index (FI) is a versatile tool for quantitatively assessing frailty, integrating a wider range of elements such as symptoms, diseases, disabilities, and laboratory values [2, 5] into a numerical score that reflects subtle changes in health status and has been associated with age and mortality [7, 8]. In emergency healthcare settings, the focus on identifying older patients at greater risk for adverse outcomes, including prolonged stays in emergency and in-patient departments, has led to a growing emphasis on early risk assessment upon patient arrival in the ED [9]. Nevertheless, a formal agreement on the optimal definition and tool for detecting frailty in the emergency department (ED) remains elusive [10]. This uncertainty extends to the practicality and effectiveness of frailty scales in the ED for risk stratification, as well as their value in prognosis [11].

In the emergency healthcare setting, the integration of specialized prognostic tools like the eFI into electronic health record (EHR) systems may facilitate immediate, precise evaluation of frailty, which can be used to design personalized care plans, ultimately improving patient outcomes [9, 12, 13]. By enabling quick and accurate frailty assessments, the eFI addresses the urgent need for tools that adapt to the dynamics of emergency medicine.

The primary aim of this study is to create a new eFI based on cumulative health and functional deficits utilizing routinely gathered clinical data by the ED physicians, thus allowing for quick and readily stratified assessment of frailty in geriatric patients presenting to the ED. The secondary aims are to evaluate the eFI performance in predicting hospital admissions, intensive care unit (ICU) admissions, and 30-day ED readmissions among geriatric patients presenting to the ED.

## Materials and methods

### Study design and participants

This study employed a retrospective approach, examining EHRs from the IRCCS Humanitas Research Hospital’s ED in Rozzano, Italy. The dataset spanned from January 2015 to December 2019 and focused on patients aged 65 and above. Exclusion criteria encompassed patients younger than 65, those who declined to participate or did not consent, individuals fast-tracked to specialized care, and cases with incomplete medical records (e.g., death upon arrival or unauthorized departures from the ED).

### Frailty assessment

The eFI was developed based on the cumulative deficit model of frailty [2, 14]. The cumulative deficit model defines frailty by considering a variety of factors such as symptoms, signs, diseases, disabilities, and abnormal laboratory values. These factors, collectively referred to as deficits, are used to identify frailty [14]. This model calculates frailty as the ratio of accumulated deficits to the total number of frailty deficits. The deficits (see eTable 1) were chosen based on a well-established frailty framework, ensuring they met the standardization criteria described by Searle and colleagues [15, 16]. Consideration was given to encompassing various organ systems and ensuring an adequate amount of critical information by including a minimum of 30 health deficits. In essence, variables utilized for a Frailty Index must be health deficits that tend to increase with age, encompassing various systems, while avoiding premature peaking. Moreover, to track frailty over time, the composition of the index should remain consistent [15].

The queried variables used to construct the eFI included laboratory values, vital parameters, and medical history and physical examination findings documented upon admission to ED or in clinical diaries, specifically within the initial 12 h following arrival. All the items taken into account were coded in a dichotomous fashion (0 = absence of the deficit, 1 = presence of the deficit). For continuous variables, we determined the range of normality based on established cut-off points found in existing literature and/or in accordance with local standards, as suggested by Patrizio et al. [17]. The eFI score ranges from 0 to 1, and it is calculated by dividing the number of deficits present by the total number of deficits collected and available. To ensure consistency, a minimum of 80% of the items must be available to calculate the eFI. Patients who did not meet the requirement of having data available for at least 80% of the 45 variables necessary for the calculation of the eFI were excluded.

For the purpose of this study, patients were divided into four quartile groups based on their eFI scores. By employing this quartile-based methodology, the study aims to provide more nuanced insights into the role of eFI as a predictor of adverse clinical outcomes. This allows for a more comprehensive understanding of how frailty, as quantified by eFI, correlates with health outcomes across a range of frailty levels. Quartile 1 consisted of patients with the lowest eFI scores, and Quartile 4 included those with the highest. The quartiles were defined as follows:

- Quartile 1: eFI score lower than 0.124; classified as non-frail
- Quartile 2: eFI score ranging from 0.125 to 0.159; classified as mildly frail
- Quartile 3: eFI score ranging from 0.159 to 0.204; classified as moderately frail
- Quartile 4: eFI score higher than 0.205; classified as severely frail

### Outcome measures

The primary outcomes were hospitalization rates, in-hospital mortality, ICU admission, and 30-day ED readmission. These outcomes were analyzed for each quartile group and compared to assess the potential association between eFI scores and adverse health outcomes.

### Statistical analysis

A *P*-value of < 0.05 was considered statistically significant. Variables were compared across the quartile groups using the chi-square test for categorical variables and the one-way ANOVA for continuous variables. Odds ratios (ORs) were computed for each quartile group, using Quartile 1 as the reference group, to assess the risk of adverse clinical outcomes. Both unadjusted and adjusted models were applied, with the latter adjusting for potential confounders such as age and sex. By employing this quartile-based methodology, the study aims to provide more nuanced insights into the role of eFI as a predictor of adverse clinical outcomes. This allows for a more comprehensive understanding of how frailty, as quantified by eFI, correlates with health outcomes across a range of frailty levels.

Baseline characteristics of the study population were displayed using either absolute and relative frequencies for categorical variables, and mean (SD) or median (IQR) for continuous variables. ORs were calculated for each of the study outcomes, including hospitalization, in-hospital death, ICU admission, and 30-day ED readmission. Both univariate and multivariate regression models, adjusted for age and sex, were applied. To explore the relationship between each outcome and eFI, logistic regression models were used for binary outcomes. Results from logistic regression are presented as ORs with 95% confidence intervals (CIs). Receiver operating characteristic (ROC) curves were computed to assess the predictive power of the frailty measures for the clinical outcomes. Statistical significance was set at *P* < 0.05. All statistical analyses were performed using STATA 17.0 SE—Standard Edition (StataCorp LLC) for Mac.

### Ethical considerations

The study titled “The use of an electronic frailty index to predict short and long-term health outcomes in geriatric patients presenting to the Emergency Department” received ethical approval from the Human Research and Ethics Committee of the IRCCS Humanitas Research Hospital (Study number: 9/23) on April 21, 2023, and was conducted in accordance with the Helsinki Declaration.

## Results

### General population overview

Our study initially analyzed data from 24,888 patients aged 65 and above. After excluding those that did not meet the 80% required for the frailty score, our final cohort comprised 21,537 patients, with a nearly even gender distribution. The cohort demographics and clinical characteristics are shown in Table 1. Of the total study population, nearly a third were hospitalized and in-hospital mortality was observed in 2.19% of cases. The overall median eFI score was 0.16 (IQR 0.13–0.20), with slight gender variations.

**Table 1:** Baseline characteristics of the general population. Values are represented as mean (standard deviation, SD) or median (interquartile range, IQR), unless otherwise stated, and as number and percentages

Variable	Value
Age (years)	77.44 (± 6.97)
Male (years)	76.87 (± 6.80)
Female (years)	78.03 (± 7.10)
Male gender	10,923 (50.7%)
Female gender	10,611 (49.3%)
Total eFI score, median (IQR)	0.16 (0.13–0.20)
eFI Males, median (IQR)	0.17 (0.13–0.21)
eFI Females, median (IQR)	0.16 (0.13–0.20)
Cardiovascular diseases	4342 (20.2%)
Lung diseases	2016 (9.36%)
Neurological diseases	1719 (7,98%)
Renal diseases	1674 (7.77%
Cerebrovascular diseases	1667 (7.74%)
Oncological diseases	1581 (7.34%)
Gastroenteric/hepatic disease	947 (4.39%)
Urologic or gynecologic disease	567 (2.63%)
Psychiatric disease	363 (1.69%)
Endocrinological disease	255 (1.18%)
1 comorbidity	10,581 (49.13%)
≥ 2 comorbidities	4086 (18.97%)
Arriving from nursing home	599 (2.78%)
Home support	401 (1.86%)
Hospitalized	7052 (32.75%)
Length of stay, mean (SD)	10.4 (± 9.34)
In-hospital mortality	472 (2.19%)
Discharged from ED	14,484 (67.25%)
ICU admission	287 (4.07%)
30-day ED readmission	3169 (14.84%)

*eFI* electronic Frailty Index, *ED* emergency department, *ICU* intensive care unit

Table 2 specifically focuses on the characteristics of patients stratified by eFI levels into quartiles.

**Table 2:** Baseline characteristics of patients stratified by electronic Frailty Index (eFI) levels into quartiles (*n* = 21,537)

eFI categories					
Variable	Quartile 1 (*n* = 5616)	Quartile 2 (*n* = 4839)	Quartile 3 (*n* = 5381)	Quartile 4 (*n* = 5700)	*P* value
Age (years)	75 (70–81)	77 (72–83)	78 (72–83)	79 (73–84)	< 0.0001
Men, *n* (%)	2630 (46%)	2401 (49%)	2760 (51%)	3140 (55%)	< 0.0001
eFI score, median (range)	0.100 (0.076, 0.113)	0.150 (0.133, 0.154)	0.1795 (0.175, 0.191)	0.2308 (0.222, 0.256)	< 0.0001
Hospitalized, *n* (%)	1163 (20%)	1367 (28%)	1883 (35%)	2439 (43%)	< 0.0001
In-hospital mortality, *n* (%)	25 (0.44%)	51 (1.1%)	110 (2.0%)	286 (5.0%)	< 0.0001
ICU admission, *n* (%)	35 (0.62%)	47 (0.97%)	81 (1.51%)	124 (2.17%)	< 0.0001
30-day ED readmission, *n* (%)	557 (9.9%)	660 (13.6%)	802 (14.9%)	1130 (19.8%)	< 0.0001

*eFI* electronic Frailty Index, *ED* emergency department, *ICU* intensive care unit

The mean age of patients increased across quartiles (*P* < 0.0001) with a similar trend observed for the proportion of men in each quartile, increasing from 46% in Quartile 1 to 55% in Quartile 4 (*P* < 0.0001). The eFI score also displayed a progressive increase across the quartiles (*P* < 0.0001). This increase in eFI was associated with higher rates of poorer outcomes. Specifically, the percentage of patients hospitalized significantly increased from Quartile 1 to Quartile 4 (*P* < 0.0001). In-hospital mortality and ICU admissions also displayed significant positive correlations with higher eFI scores. Mortality rates increased from 0.44% in Quartile 1 to 5.0% in Quartile 4 (*P* < 0.0001). ICU admissions followed a similar trend, rising from 0.62% in Quartile 1 to 2.17% in Quartile 4 (*P* < 0.0001). The 30-day ED readmission rates exhibited a similar pattern, showing a significant increase from Quartile 1 to Quartile 4 (*P* < 0.0001).

**Fig. 1:**
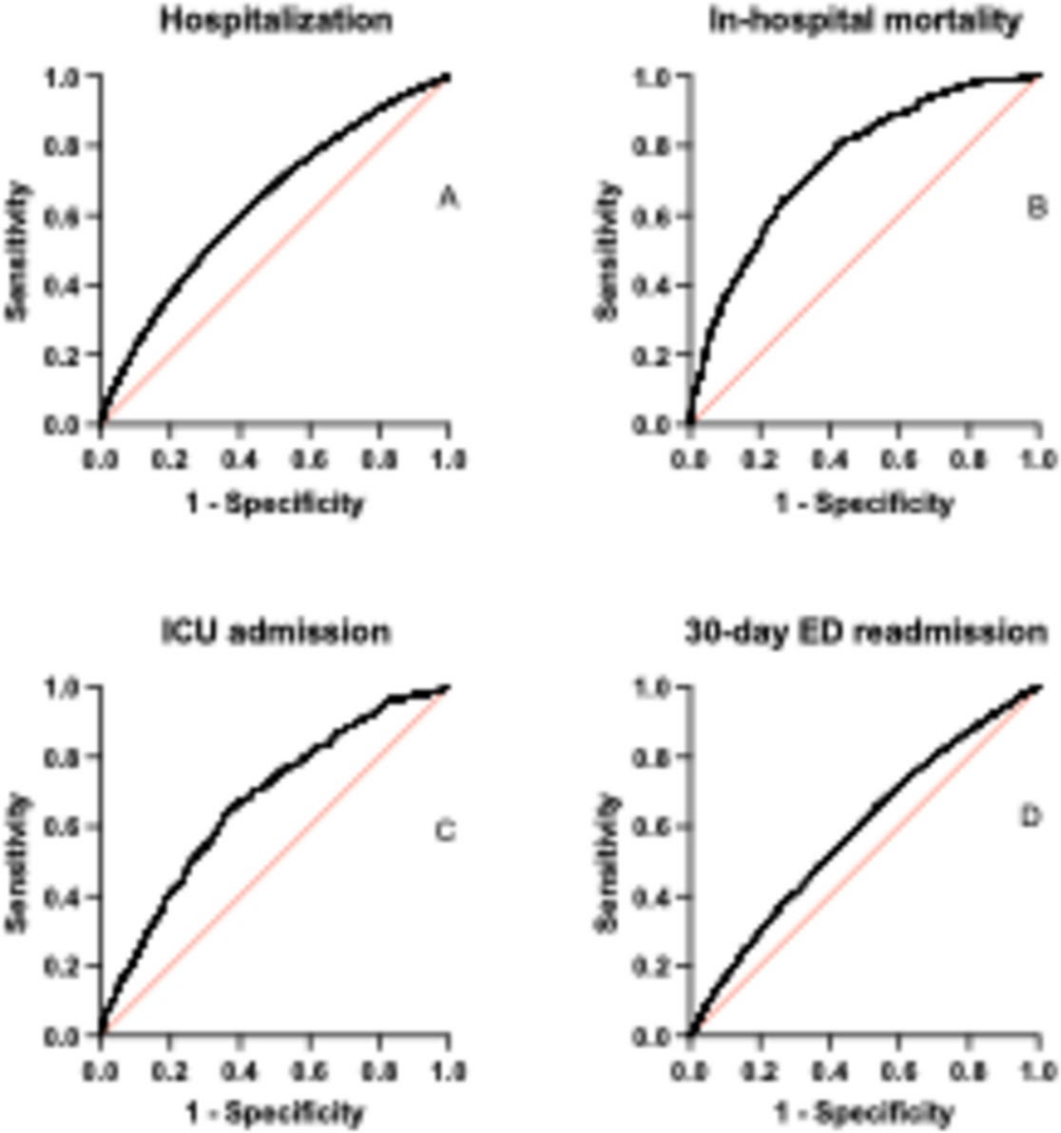
ROC analysis, adjusted for age and gender, of eFI as a continuous variable with regards to hospitalization (**A**), in-hospital mortality (**B**), ICU admission (**C**), and 30-day ED readmission (**D**)

Table 3 elaborates on the association between eFI and various clinical outcomes, both unadjusted and adjusted for age and sex. For hospitalization, the odds ratio (OR) per 0.01 increase in eFI score was 1.34 unadjusted and 1.35 adjusted (both *P* < 0.0001). These findings were supported by a moderate area under the curve (AUC) of 0.6361 (adjusted) indicating fair predictive accuracy (see Fig. 1A and Table 3). When hospitalization was examined across eFI quartiles, the risk significantly increased from Quartile 1 (reference) to Quartile 4 (see Table 3). For in-hospital mortality, each 0.01 increase in eFI score corresponded with a significant increase in the odds of mortality. A high AUC of 0.7534 (adjusted) supported the model’s robustness (see Fig. 1B and Table 3). Adjusted OR in Quartile 4 reached 14.15, which was highly significant (see Table 3). Regarding ICU admission, both unadjusted and adjusted ORs indicated a statistically significant increase for each 0.01 increase in eFI score, supported by a moderate AUC of 0.6714 (adjusted) (see Fig. 1C and Table 3). Among quartiles, the risk rose significantly, with an adjusted OR of 4.70 in Quartile 4 (see Table 3). For 30-day ED readmission, the unadjusted and adjusted ORs per 0.01 increase in eFI were 1.21 and 1.20, respectively (both *P* < 0.0001). These findings were supported by a mild AUC of 0.5856 (adjusted) (see Fig. 1D and Table 3). Across quartiles, the adjusted OR in Quartile 4 was 2.22 (*P* < 0.0001) (see Table 3).

**Table 3:** Association of electronic Frailty Index with hospitalization, in-hospital mortality, ICU admission, and 30-day ED readmission

	**Unadjusted**		**Adjusted for age and sex**	
	OR (95%CI)	AUC	OR (95%CI)	AUC
Hospitalization				
Continuous variable	1.34 (1.19–1.51)***	0.6313	1.35 (1.20–1.52)***	0.6361
Quartile 1	1 (Reference)		1 (Reference)	
Quartile 2	1.51 (1.38–1.65) ***	0.5511	1.54 (1.36–1.76) ***	0.5719
Quartile 3	2.06 (1.89–2.25) ***	0.5891	2.05 (1.88–2.24) ***	0.6080
Quartile 4	3.30 (3.04–3.59) ***	0.6434	3.55 (3.15–4.01) ***	0.6610
In-hospital mortality				
Continuous variable	1.63 (1.42–1.91) ***	0.7490	1.60 (1.40–1.88) ***	0.7534
Quartile 1	1 (Reference)		1 (Reference)	
Quartile 2	2.38 (1.49–3.91) **	0.6049	2.61 (1.19–6.16) *	0.6503
Quartile 3	4.67 (3.07–7.38) ***	0.6648	5.41 (2.77–11.79) ***	0.7004
Quartile 4	11.81 (8.01–18.26) ***	0.7138	14.15 (7.92–28.11) ***	0.7332
ICU admission				
Continuous variable	1.38 (1.25–1.59) ***	0.6424	1.42 (1.29–1.60) ***	0.6714
Quartile 1	1 (Reference)		1 (Reference)	
Quartile 2	1.56 (1.01–2.44) *	0.5556	1.58 (0.81–3.13)	0.5996
Quartile 3	2.44 (1.65–3.67) ***	0.6056	2.71 (1.47–5.21) **	0.6691
Quartile 4	3.55 (2.46–5.24) ***	0.6400	4.70 (2.73–8.57) ***	0.6857
30-day ED readmission				
Continuous variable	1.21 (1.15–1.29) ***	0.5840	1.20 (1.14–1.28) ***	0.5856
Quartile 1	1 (Reference)		1 (Reference)	
Quartile 2	1.38 (1.23–1.55) ***	0.5401	1.31 (1.11–1.56) **	0.5529
Quartile 3	1.53 (1.37–1.72) ***	0.5527	1.57 (1.34–1.85) ***	0.5594
Quartile 4	2.16 (1.94–2.41) ***	0.5932	2.22 (1.90–2.60) ***	0.6014

*ED* emergency department, *ICU* intensive care unit

^*^P < 0.05

^**^P < 0.01

^***^P < 0.001

## Discussion

Our thorough research of 21,537 individuals aged 65 and beyond sheds light on the association between frailty, as measured by the eFI, and clinical outcomes in older adults. The balanced gender distribution and wide spectrum of common disorders such as cardiovascular, pulmonary, neurological, renal, and cerebrovascular problems, with almost half of patients having at least one comorbidity, make the study relevant to a normal senior population. A key conclusion is that higher eFI scores increase hospitalization, in-hospital mortality, ICU admissions, and 30-day ED readmissions. The odds ratios for these outcomes, both unadjusted and adjusted for age and sex, showed a significant risk increase with each eFI score increase, validating the index’s usefulness as a predictor of unfavorable health outcomes.

The stratification of patients into quartiles based on eFI scores revealed a clear trend; with the increasing frailty level, there was a corresponding increase in the mean age and the proportion of male patients. This trend was particularly pronounced in the highest quartile, which also reported the worst clinical outcomes. These findings echo the results from previous studies, which consistently show frailty to be more prevalent in older populations [18–20]. Moreover, the higher proportion of men in the upper quartiles correlates with past research showing that frail men may be at higher risk for adverse outcomes than their female counterparts [21]. The stark contrast in hospitalization rates, from 20% in the least frail quartile to 43% in the most frail, together with similar mortality and ICU admission patterns, highlights the progressive nature of frailty and its impact on healthcare resource utilization. In-hospital death rates increased approximately fivefold from Quartile 1 to Quartile 4, indicating a need for preventative measures or closer monitoring for patients in the higher eFI quartiles. The rising rate of ICU admissions with greater eFI levels suggests frailer patients need specialized care. ICU resources are often limited, and knowing that a patient has a higher eFI score could lead to earlier interventions that might prevent the need for ICU admission altogether. Similarly, the sharp rise in 30-day ED readmission rates across eFI quartiles suggests that the eFI could be an essential tool for identifying patients at higher risk for readmission, thereby helping to tailor discharge plans and post-hospitalization support.

The progressively higher odds ratios, even after adjusting for age and sex, for hospitalization, in-hospital mortality, ICU admission, and 30-day ED readmission across eFI quartiles highlight the score’s utility for risk stratification and align well with studies such as Searle et al. [15], which found a similar upward trend. Of particular concern is the substantial rise in in-hospital mortality rates and ICU admissions in the higher eFI quartiles. The adjusted OR for in-hospital mortality in Quartile 4 was exceptionally high, emphasizing the urgency of targeted interventions for this subgroup. This finding coincides with findings from recent studies highlighting the value of Frailty Indexes in predicting mortality [8, 22]. One of the more striking findings was the association between eFI and ICU admissions. While limited research has been focused on this aspect, a study by Bagshaw et al. [23] highlighted that frailty could serve as an independent predictor for ICU admission. The association between hospital frailty and 30-day ED readmission was also reported by previous studies [24–26].

In evaluating the outcomes of our study, particularly the “relative weakness” observed in 30-day ED readmission and hospitalization rates compared to more severe outcomes such as ICU admission and in-hospital mortality, it is crucial to consider the role of social factors. These factors, beyond the clinical indicators of frailty, may significantly influence patient management decisions. Specifically, the decision to hospitalize a patient or their return to the ED may be based not just on clinical frailty, but also on their amount of social support. This suggests that the presence or absence of social support can influence whether a patient is sent home or hospitalized, potentially leading to a higher risk of readmission to the ED for those lacking adequate support. Recognizing this, future research should include social aspects in frailty-related outcome studies to provide a more comprehensive picture of patient management dynamics, particularly in the context of ED readmissions and hospitalization decisions.

Applying the eFI in the ED has the potential to significantly enhance patient care through personalized interventions. While our study did not directly investigate these specific outcomes, the capabilities of the eFI suggest it could be instrumental in identifying individuals who may benefit more from in-depth evaluation and management to solve possible reversible causes of frailty before proceeding with standard interventions. For patients with elevated eFI scores, the index might be helpful in formulating comprehensive care interventions. These plans could include initiating home health care services, arranging for rehabilitation in assisted living facilities or nursing homes, organizing physiotherapy sessions, and ensuring regular follow-up appointments. Moreover, the potential creation of multidisciplinary care teams, comprising social workers, doctors, nurses, and physiotherapists, could provide continuous, coordinated care. These interventions are hypothesized to speed up the recovery process and reduce the chances of readmission to the ED, highlighting a promising area for future research to explore the practical impacts of the eFI in clinical settings.

The utility of an automated frailty assessment tool, such as the eFI, in EDs becomes increasingly evident, especially when considering the limitations of manual screening methods. A study by Blomaard et al. [27] highlights these limitations, revealing a significant decrease in the likelihood of conducting manual frailty assessments during busy times and with severely ill patients. This points to the vulnerability of traditional methods to the dynamic and high-pressure environment of EDs. Unlike these conventional tools, the eFI usage of advanced analytics integrated within existing EHR systems to provide rapid real-time assessments of a patient’s frailty level. This capability is particularly beneficial in the fast-paced environment of the ED where time is critical and comprehensive manual frailty assessment may not be feasible [28]. In addition, using data from EHRs make the frailty assessment more objective and less prone to the variability and subjectivity of clinical judgment or patient self-reporting [29]. This objectivity is crucial in the ED, where decisions often need to be made rapidly and based on the most reliable information available. Furthermore, the eFI’s design facilitates seamless integration with existing healthcare IT infrastructures, enhancing the feasibility of ongoing monitoring and management in large healthcare settings. This integration may not only lead to improved efficiency in healthcare delivery, with automated alerts and updates that may aid in timely decision-making but it could also ensure that healthcare providers always have access to the latest insights when making critical decisions.

### Limitations

The current study, being confined to a single center, presents certain limitations that must be acknowledged. Firstly, there is a need for both internal and external validation to ensure the robustness and applicability of our findings. Additionally, our eFI has not been validated against other clinical eFIs for the same cohort, which is a crucial step for confirming its reliability and comparability. Another limitation is the demographic focus of the study. The eFI was developed based on data from hospitalized populations aged 65 and above, as this age group predominantly represents frail older individuals in Western Europe. Consequently, our findings and the validity of the score may not be applicable to younger populations, specifically those under the age of 65. This restricts the generalizability of our eFI and suggests that further research is needed to explore its relevance and effectiveness in younger age groups.

## Conclusions

This study concludes that the eFI has the potential to identify older patients at risk of hospitalization, in-hospital death, ICU admission, and ED readmissions. Its use in emergency care settings may improve risk assessment and resource allocation, potentially improving patient outcomes in high-pressure conditions. Specifically, the eFI could facilitate the early initiation of tailored therapies for elderly patients, who are especially vulnerable in the fast-paced ED environment.

Future studies should validate the eFI in EDs to determine its applicability and efficacy. Further research is crucial to confirm these preliminary findings and explore their integration into routine clinical workflows. The eFI’s relevance and value in emergency care will depend on its continual refinement and assessment to meet the demands of older patients in critical care settings.

## Supplementary Information

Below is the link to the electronic supplementary material.

Supplementary file1 (DOCX 20 KB)

## Data Availability

Data is available upon request.
