# Supplementary material for: Enhancing emergency care for older persons: the role and impact of the electronic Frailty Index
Source: GeroScience. 2025 Jan 13;47(3):3207–15. doi: 10.1007/s11357-024-01472-x (PMC12181595; doi:10.1007/s11357-024-01472-x)
Supplement: Supplementary file 1 — Supplementary file1 (DOCX 20 KB) [file 11357_2024_1472_MOESM1_ESM.docx]

Table A. Variables (n=45) used to compute the eFI.

| VARIABLES / DEFICITS | CUT-POINTS |
| --- | --- |
| Respiratory rate | No ≤ 20 BPM; Yes > 20 BPM |
| Oxygen saturation alteration | No ≥ 90%; Yes < 90% |
| Systolic blood pressure alteration | No = 90-130mmHg; Yes < 90 or > 130 mmHg |
| Diastolic blood pressure alteration | No = 51-90mmHg; Yes ≤ 50 or > 90mmHg |
| Heart rate alteration | No = 51-100bpm; Yes ≤ 50 or > 100bpm |
| Body temperature | 0 < 37°C; 1 ≥ 37°C |
| Glasgow Coma Score | No = 9-15; Yes ≤ 8 |
| Polypharmacy (>5 drugs) | No = 0; Yes = 1 |
| Use of sedative, hypnotic and/or neuroleptic drugs | No = 0; Yes = 1 |
| Poor hygienic conditions | No = 0; Yes = 1 |
| Acute pain | No = 0; Yes = 1 |
| Agitation | No = 0; Yes = 1 |
| Disorientation | No = 0; Yes = 1 |
| Constipation | No = 0; Yes = 1 |
| Patient coming from nursing home | No = 0; Yes = 1 |
| Patient requires assistance with daily activities | No = 0; Yes = 1 |
| Vision impairment | No = 0; Yes = 1 |
| Chronic Pain | No = 0; Yes = 1 |
| Diabetes mellitus | No = 0; Yes = 1 |
| Cardiovascular disease | No = 0; Yes = 1 |
| Cerebrovascular disease | No = 0; Yes = 1 |
| Lung disease | No = 0; Yes = 1 |
| Neurological disease | No = 0; Yes = 1 |
| Psychiatric disease, mood disorder | No = 0; Yes = 1 |
| Oncological or hematological disease | No = 0; Yes = 1 |
| Renal disease | No = 0; Yes = 1 |
| Urological or gynecological disease | No = 0; Yes = 1 |
| Gastroenteric or hepatic disease | No = 0; Yes = 1 |
| Endocrinological disease | No = 0; Yes = 1 |
| Ear, Neck, Throat disease | No = 0; Yes = 1 |
| Dermatological disease | No = 0; Yes = 1 |
| Abdominal exam abnormalities | No = 0; Yes = 1 |
| Cardiovascular exam abnormalities | No = 0; Yes = 1 |
| Thoracic exam abnormalities | No = 0; Yes = 1 |
| Cutaneous exam abnormalities | No = 0; Yes = 1 |
| Neurological exam abnormalities | No = 0; Yes = 1 |
| Potassium alteration | No = 3.6-5.0 mEq/L; Yes ≤ 3.5 or > 5.0 mEq/L |
| Glucose abnormality | No = 61-126 mg/dL; Yes > 126 or ≤ 60 mg/dL |
| Creatinine abnormality | No ≤ 1.2 mg/dL; Yes > 1.2mg/dL |
| Hemoglobin abnormality | No = within reference range; Yes = < reference range |
| White blood cell abnormality | No = 4,001-10,000/mmc; Yes ≤ 4,000 or > 10,000/mmc |
| Platelets count abnormality | No = 150,001-450,000/mmc; Yes ≤ 150,000 or > 450,000/ mmc |
| Hematocrit abnormality | No = within reference range; Yes < or > than reference range |
| Alanine transaminase abnormality | No ≤ 35 UI/L; Yes > 35 UI/L |
| Aspartate transaminase abnormality | No ≤ 35 UI/L; Yes > 35 UI/L |

The eFI utilised in this study is produced from electronic health records of patients who presented to our emergency department (ED). These data were collected by nurses and ED physicians within the first twelve hours of the patient accessing the ED. These records included the patient's vital parameters, medical history, physical examination findings, medical notes, and laboratory results. Specific keywords used daily by the medical practitioners were identified for each deficit being analyzed. These keywords were the foundation for the data extraction.

All the items taken into account were coded in a dichotomous fashion (0= absence of the deficit, 1= presence of the deficit). For continuous variables, we determined the range of normality based on traditional cut-off points found in existing literature and/or in line with local standards.

The eFI ranged from 0 to 1 and was calculated by the number of deficits present divided by the total number of deficits (n=45). A cut-off of at least the 80% of the items was required to calculate the eFI to ensure consistency.
